# Supplementary material for: acorde unravels functionally interpretable networks of isoform co-usage from single cell data
Source: Nat Commun. 2022 Apr 5;13:1828. doi: 10.1038/s41467-022-29497-w (PMC8983708; doi:10.1038/s41467-022-29497-w)
Supplement: Supplementary file 1 — Supplementary Information [file 41467_2022_29497_MOESM1_ESM.pdf]

acorde unravels functionally interpretable networks of isoform co-usage from single cell data

Supplementary Figures

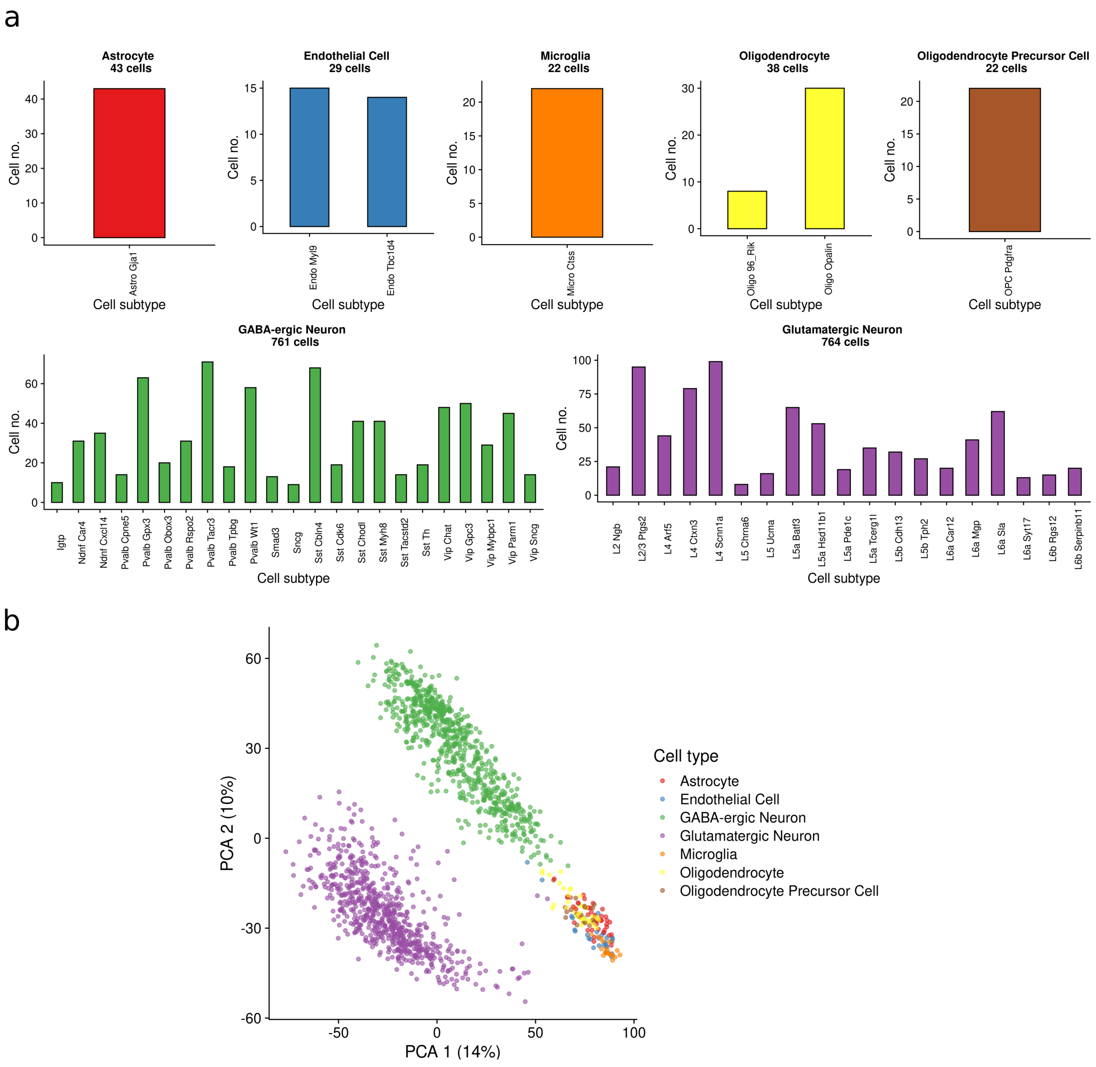

Supplementary Fig. 1 Tasic et al. mouse neural dataset overview. a Number of cells assigned to each cell type and subtype by Tasic et al. (post-QC, 1591 cells). **b** Principal Component Analysis of the full dataset, components 1 (x-axis) and 2 (y-axis) are shown.

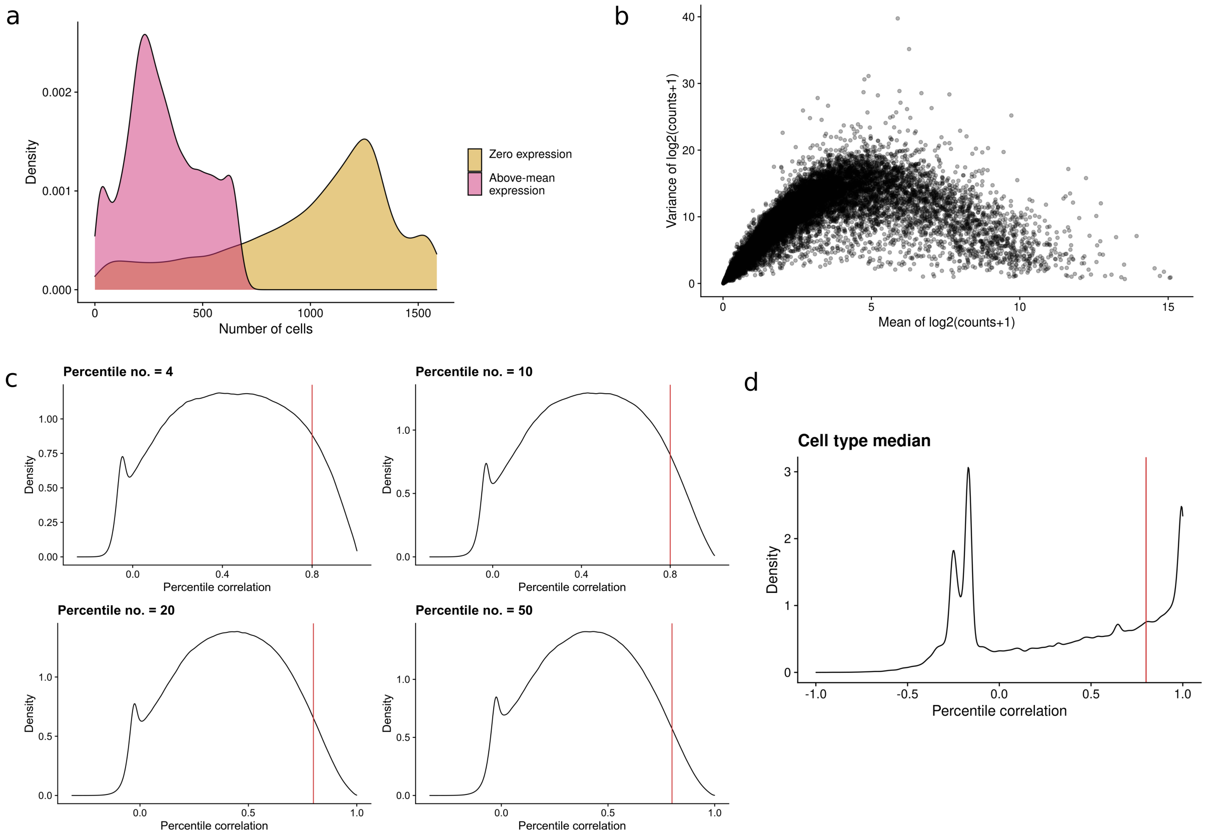

Supplementary Fig. 2 Impact of Tasic dataset properties on percentile correlations. a Heterogeneity and sparsity in the Tasic et al. dataset. x-axis shows number of cells per isoform that have zero expression (yellow distribution) or expression higher than the isoform mean (pink distribution). y-axis shows density. **b** Dataset mean-variance relationship. Dots represent transcript isoforms, x-axis represents isoform mean expression (log2counts+1) and y-axis corresponds to isoform expression variance (log2counts+1). **c** Density distribution of pairwise isoform percentile correlations obtained using different number of percentiles (i.e. 4, 10, 20 and 50). **d** Density distribution of pairwise Pearson correlation obtained using median cell type-level expression (percentile no. = 1).

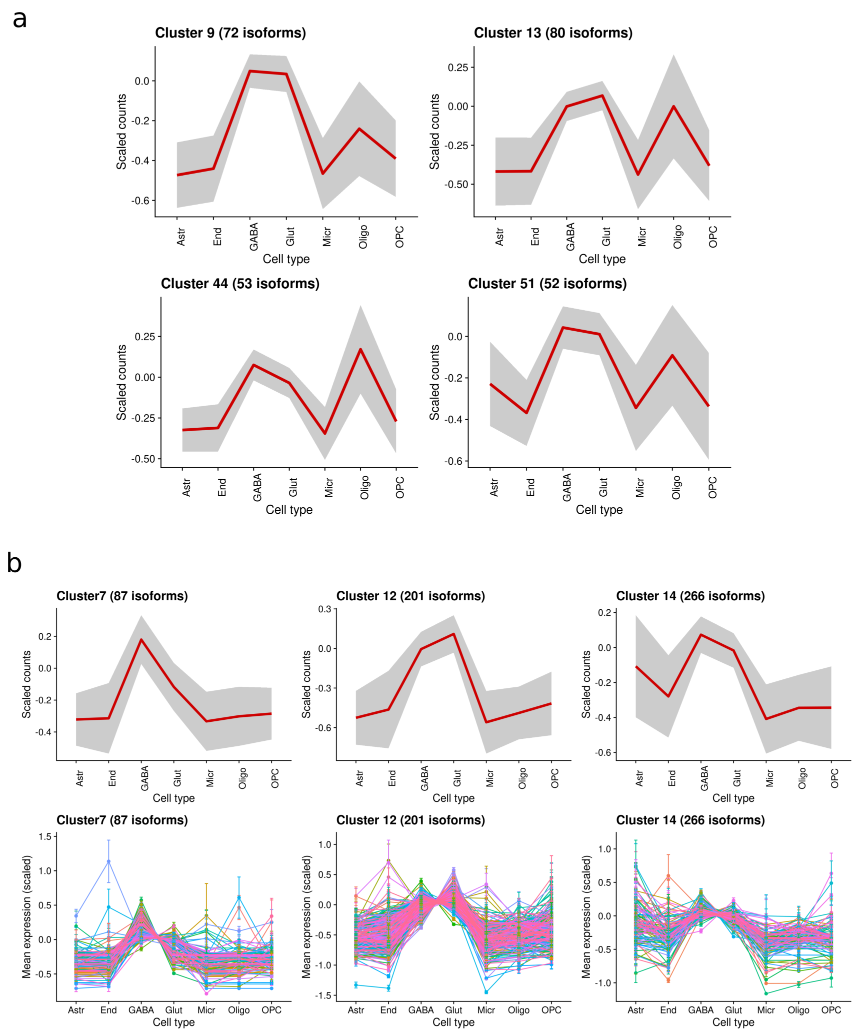

Supplementary Fig. 3 Cluster refinement examples. a Example of four redundant clusters, i.e. clusters representing the same expression profiles, that were merged by profile similarity. **b** Example of noisy clusters, i.e. clusters grouping isoforms with highly dissimilar expression patterns across cell types, whose isoforms were re-assigned to other clusters. For the single-line plots (a and b), cell-level mean expression (scaled, see Methods) is computed for all transcripts and then aggregated as the global cell type mean, represented by the red line. Grey area corresponds to cell type mean ± standard deviation. For the multi-line plots (lower panel in b), each line corresponds to the cell-type mean expression values of one transcript and the error bar corresponds to ± standard deviation, that is, the standard deviation of transcript-level mean values in each cell type. In this case, n corresponds to the number of cells per cell type, i.e. Astr: 43 cells, End: 29 cells, GABA: 761 cells, Glut: 764 cells, Micr: 22 cells, Oligo: 38 cells, OPC: 22 cells. Astr: astrocytes, End: endothelial cells, GABA: GABA-ergic neurons, Glut: glutamatergic neurons, Micr: microglia, Oligo: oligodendrocytes, OPC: oligodendrocyte precursor cells.

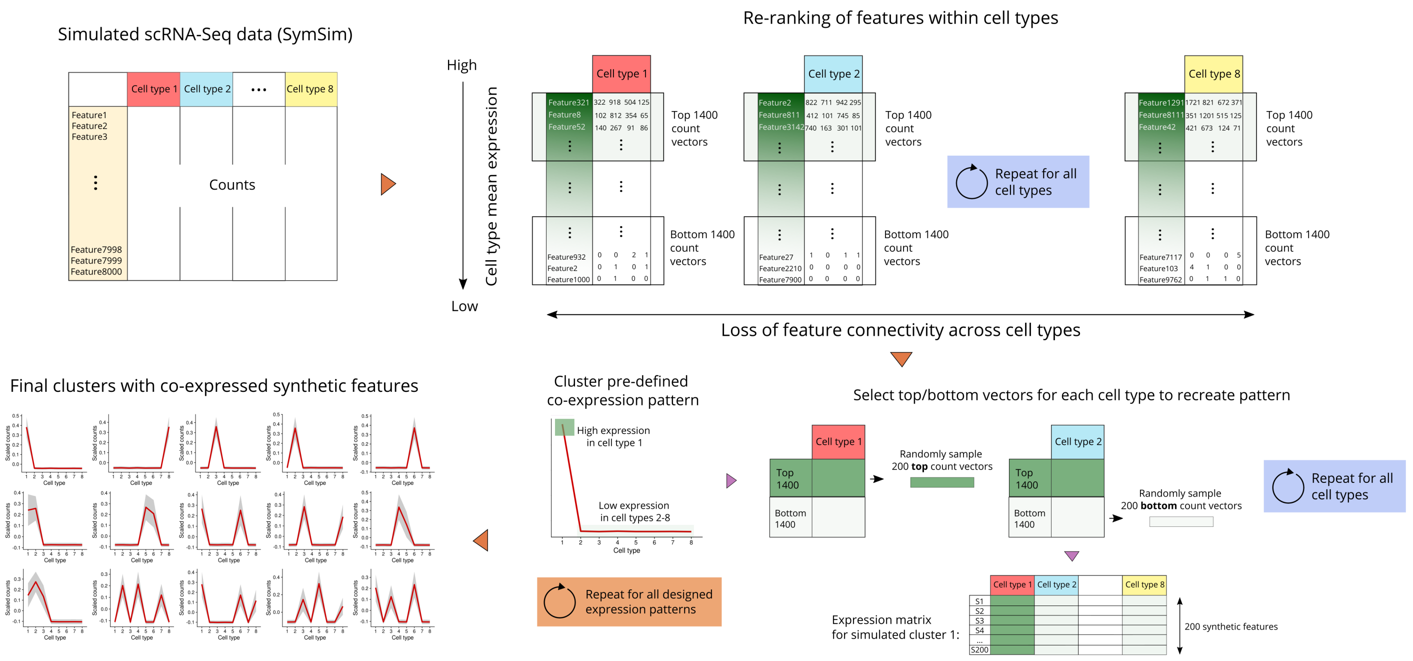

Supplementary Fig. 4 Simulation strategy used to generate single-cell co-expression patterns. After simulating single-cell RNA-seq data with SymSim, features were re-ranked within each cell type, using the simulated expression values to generate groups of co-expressed transcripts. Simulated clusters were generated according to 15 pre-defined patterns.

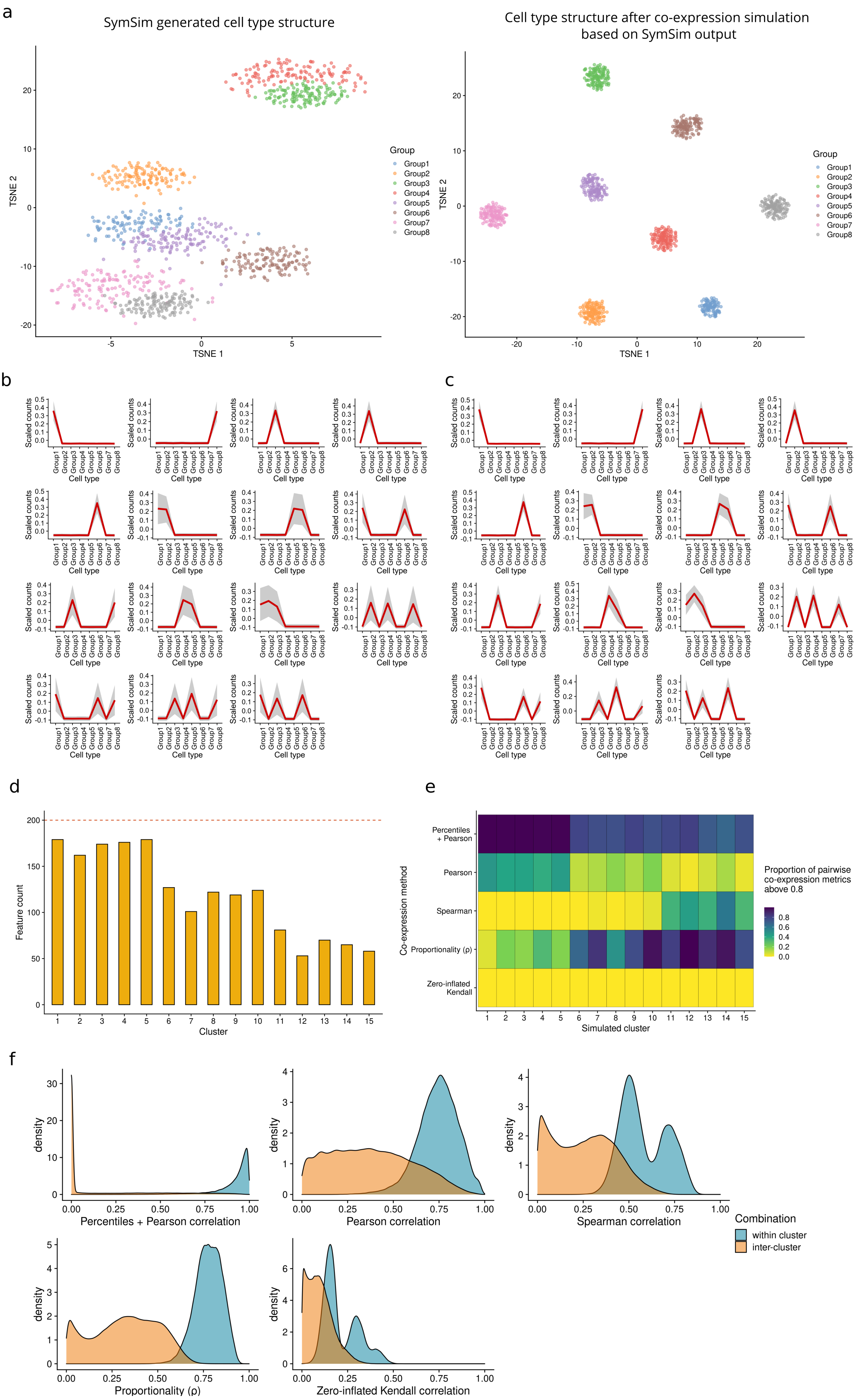

a
SymSim generated cell type structure
Cell type structure after co-expression simulation
based on SymSim output
TSNE 1
TSNE 2
Group
Group1
Group2
Group3
Group4
Group5
Group6
Group7
Group8
b
c
Scaled counts
Cell type
d
Feature count
Cluster
e
Co-expression method
Percentiles + Pearson
Pearson
Spearman
Proportionality (ρ)
Zero-inflated Kendall
Simulated cluster
Proportion of pairwise co-expression metrics above 0.8
f
density
Percentiles + Pearson correlation
Pearson correlation
Spearman correlation
Proportionality (ρ)
Zero-inflated Kendall correlation
Combination
within cluster
inter-cluster

Supplementary Fig. 5 Simulated data characterization. a Left: cell type structure of SymSim-simulated single-cell RNA-Seq data (left) depicted using t-SNE. Right: cell type structure after performing co-expression pattern simulation on the simulated counts (t-SNE). Cell grouping is effectively preserved after artificially creating synthetic, co-expressed features using simulated feature counts. **b** and **c** Expression profile of the 15 simulated clusters obtained, before (b) and after (c) filtering synthetic features showing dissimilarity with the simulated profile. Cell-level mean expression (scaled, see Methods) is computed for all simulated transcripts in the cluster and then aggregated as the global cell type mean, represented by the red line. Grey area corresponds to cell type mean ± standard deviation. **d** Number of transcripts in simulated clusters, calculated after filtering clusters to ensure cluster profile consistency. **e** Heatmap representing the proportion of high co-expression values (correlation > 0.8), computed for all pairs of synthetic transcripts within each simulated cluster. Darker colors indicate successful recapitulation of simulated co-expression relationships by the evaluated co-expression metrics. **f** Correlation density distributions obtained when computing pairwise correlations between all possible within-cluster and between-cluster synthetic feature pairs, considering the simulated clusters. Results are shown for all evaluated co-expression metrics.

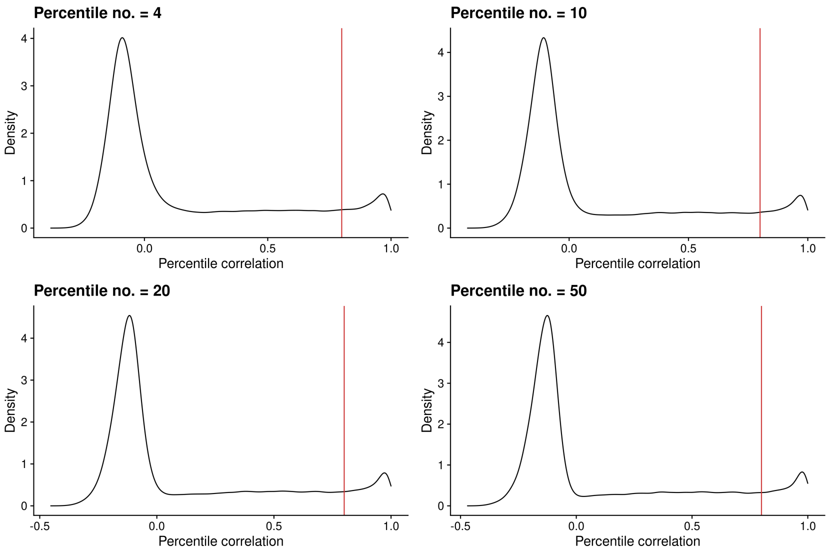

Supplementary Fig. 6 Effect of percentile number in simulated data. Percentile correlation density distributions obtained after using different number of percentiles to summarize cell type-level expression.

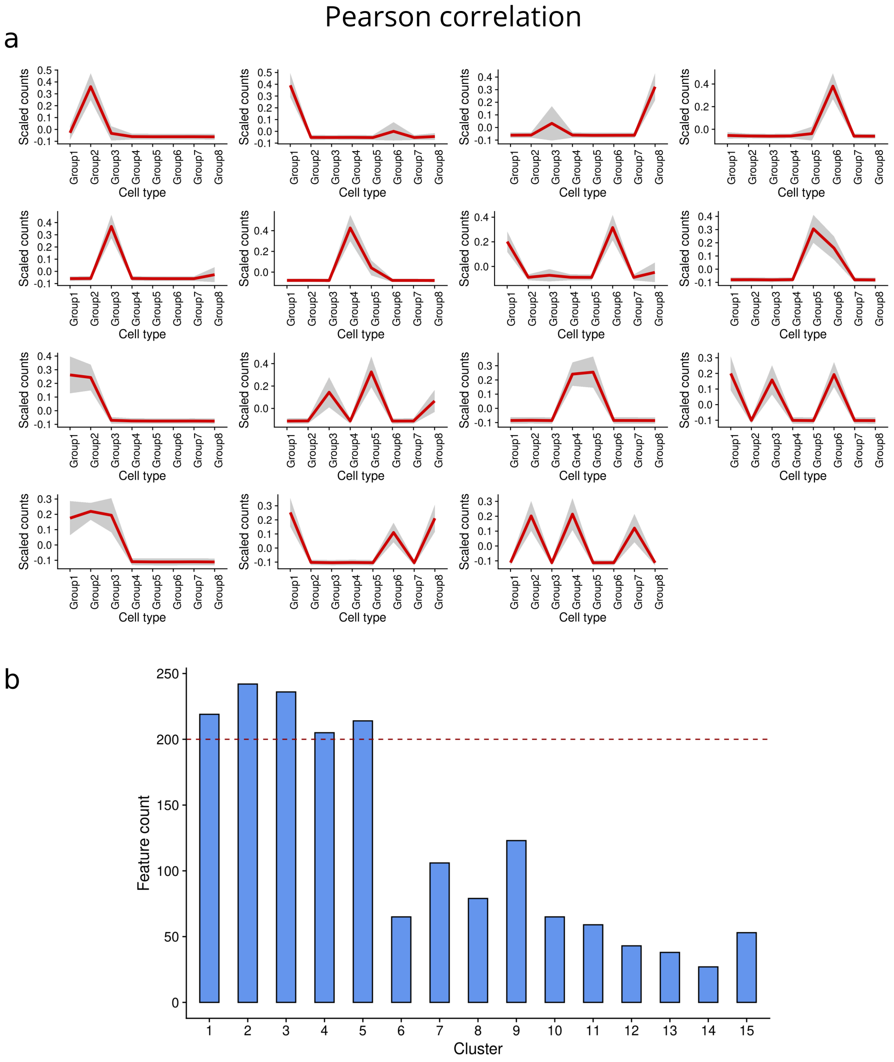

Supplementary Fig. 7 Clustering of simulated single-cell data using Pearson correlation as distance. a Mean profiles of generated clusters. Cell-level mean expression (scaled, see Methods) was computed for all simulated transcripts in the cluster and then aggregated as the global cell type mean, represented by the red line. Grey area corresponds to cell type mean ± standard deviation. **b** Number of simulated transcripts per cluster.

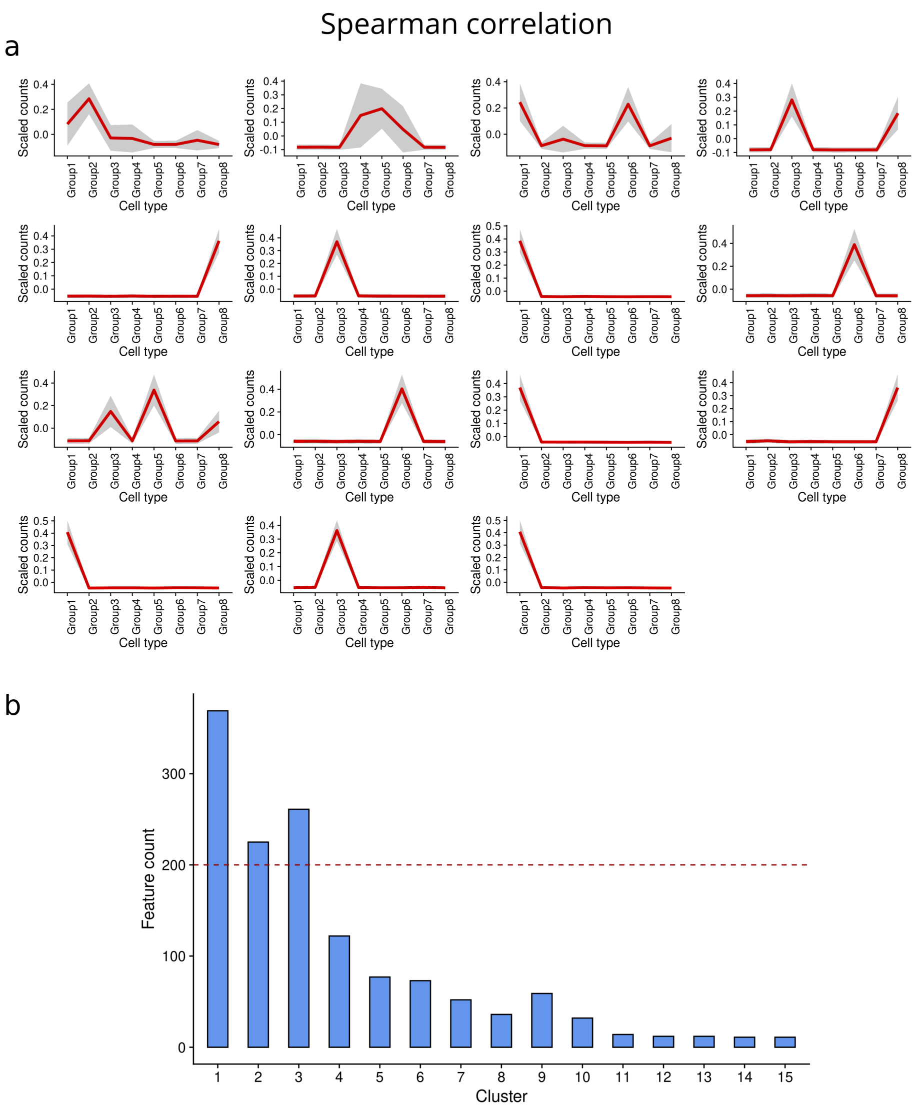

Supplementary Fig. 8 Clustering of simulated single-cell data using Spearman correlation as distance. a Mean profiles of generated clusters. Cell-level mean expression (scaled, see Methods) was computed for all simulated transcripts in the cluster and then aggregated as the global cell type mean, represented by the red line. Grey area corresponds to cell type mean ± standard deviation. **b** Number of simulated transcripts per cluster.

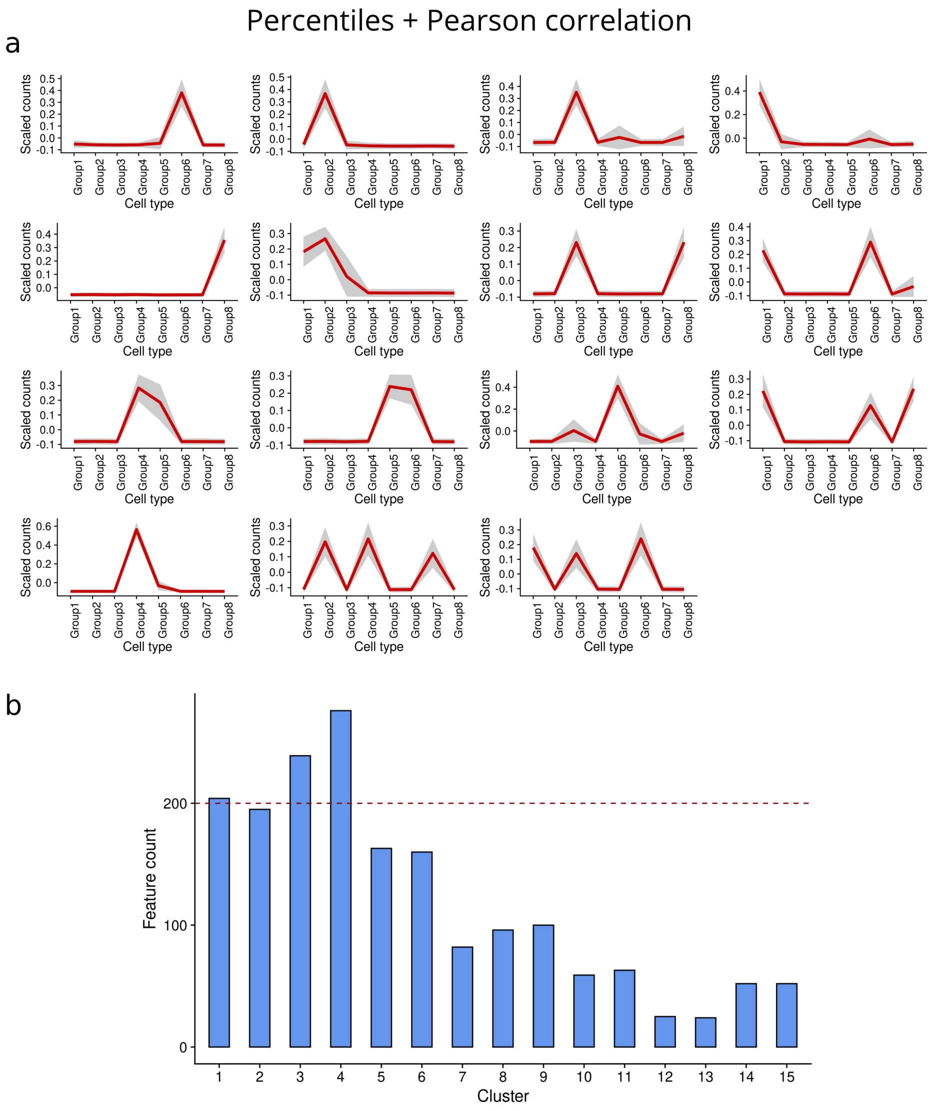

Supplementary Fig. 9 Clustering of simulated single-cell data using percentiles + Pearson correlation as distance. a Mean profiles of generated clusters. Cell-level mean expression (scaled, see Methods) was computed for all simulated transcripts in the cluster and then aggregated as the global cell type mean, represented by the red line. Grey area corresponds to cell type mean ± standard deviation. **b** Number of simulated transcripts per cluster.

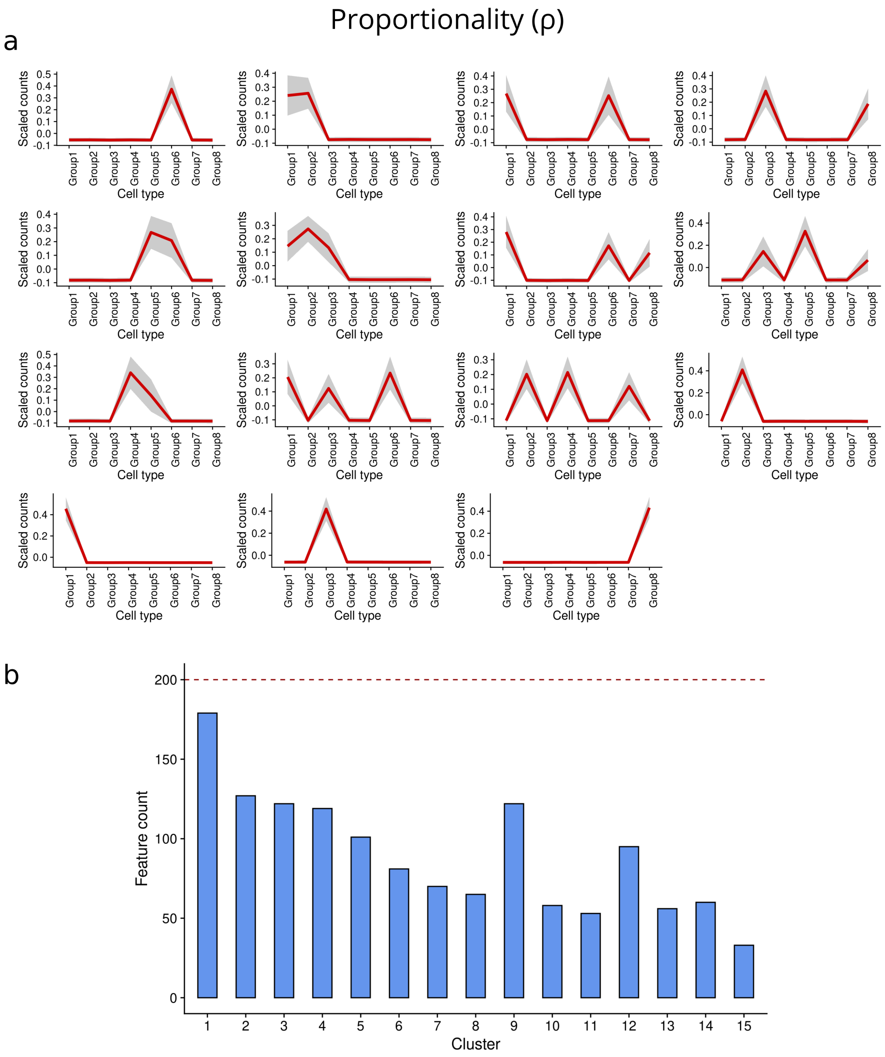

Supplementary Fig. 10 Clustering of simulated single-cell data using proportionality (ρ) as distance. a Mean profiles of generated clusters. Cell-level mean expression (scaled, see Methods) was computed for all simulated transcripts in the cluster and then aggregated as the global cell type mean, represented by the red line. Grey area corresponds to cell type mean ± standard deviation. **b** Number of simulated transcripts per cluster.

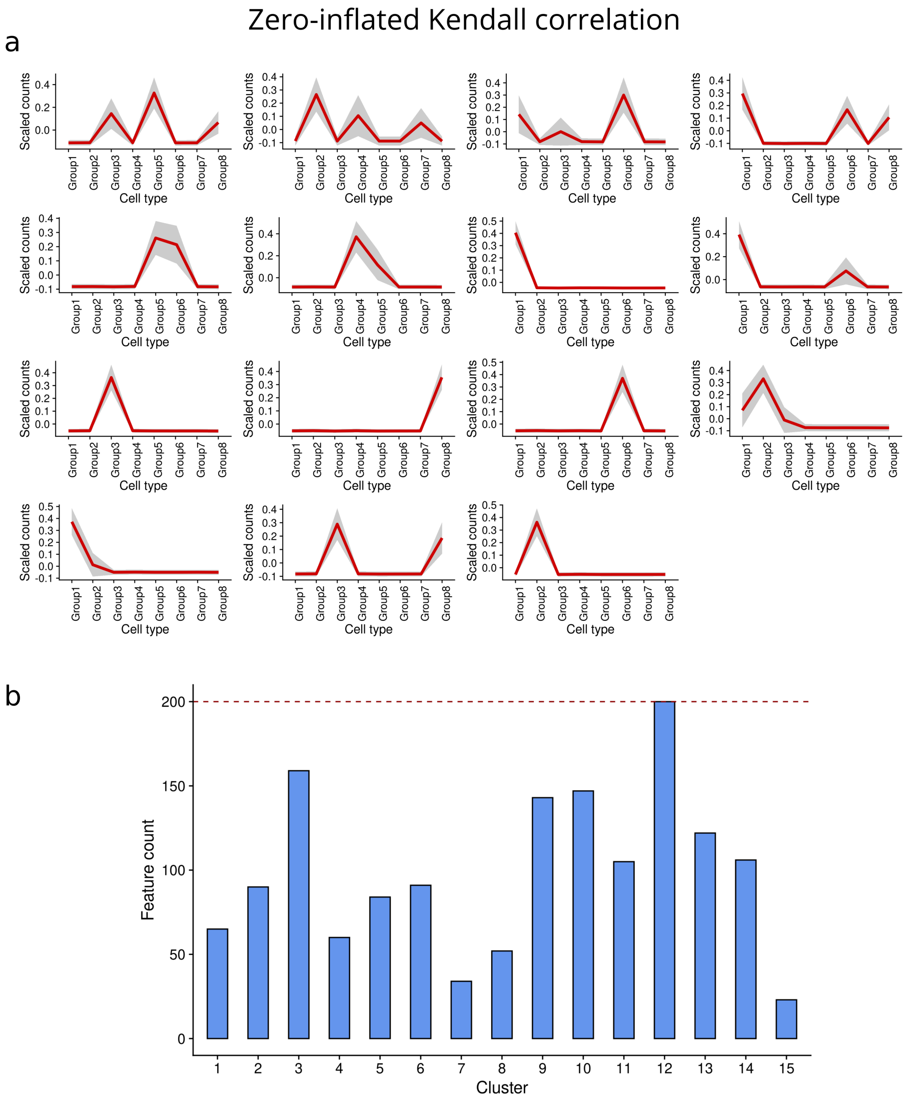

Supplementary Fig. 11 Clustering of simulated single-cell data using zero-inflated Kendall correlation as distance. a Mean profiles of generated clusters. Cell-level mean expression (scaled, see Methods) was computed for all simulated transcripts in the cluster and then aggregated as the global cell type mean, represented by the red line. Grey area corresponds to cell type mean ± standard deviation. **b** Number of simulated transcripts per cluster

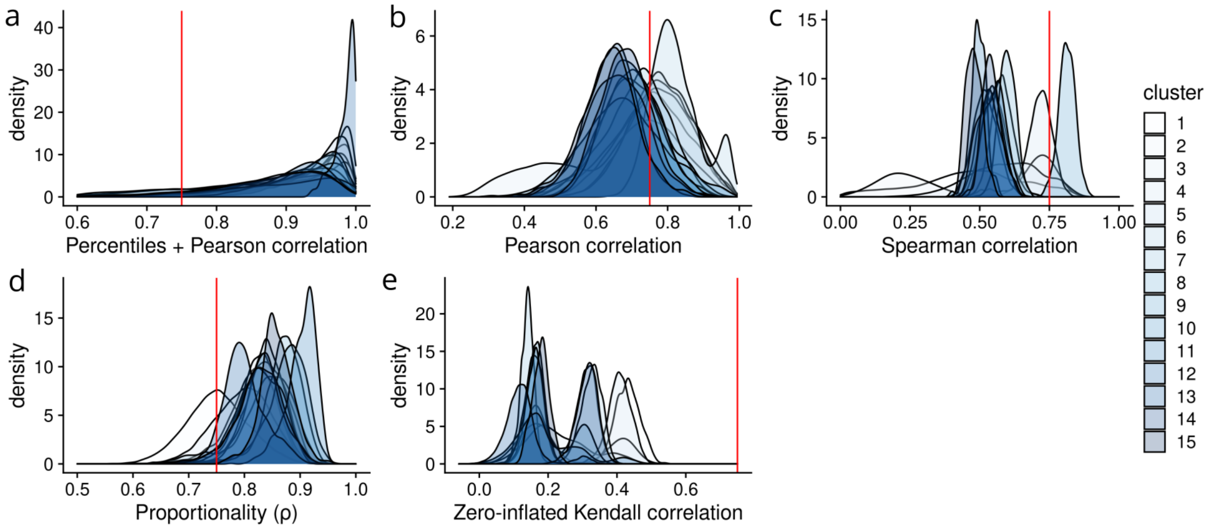

Supplementary Fig. 12 Density distributions of pariwise correlation values computed between isoforms in the simulated clusters. The red line indicates the 0.75 threshold value. Results shown for the clusters generated using each evaluated metric: **a** Percentiles + Pearson correlation. **b** Pearson correlation. **c** Spearman correlation. **d** Proportionality (ρ). **e** Zero-inflated Kendall correlation.

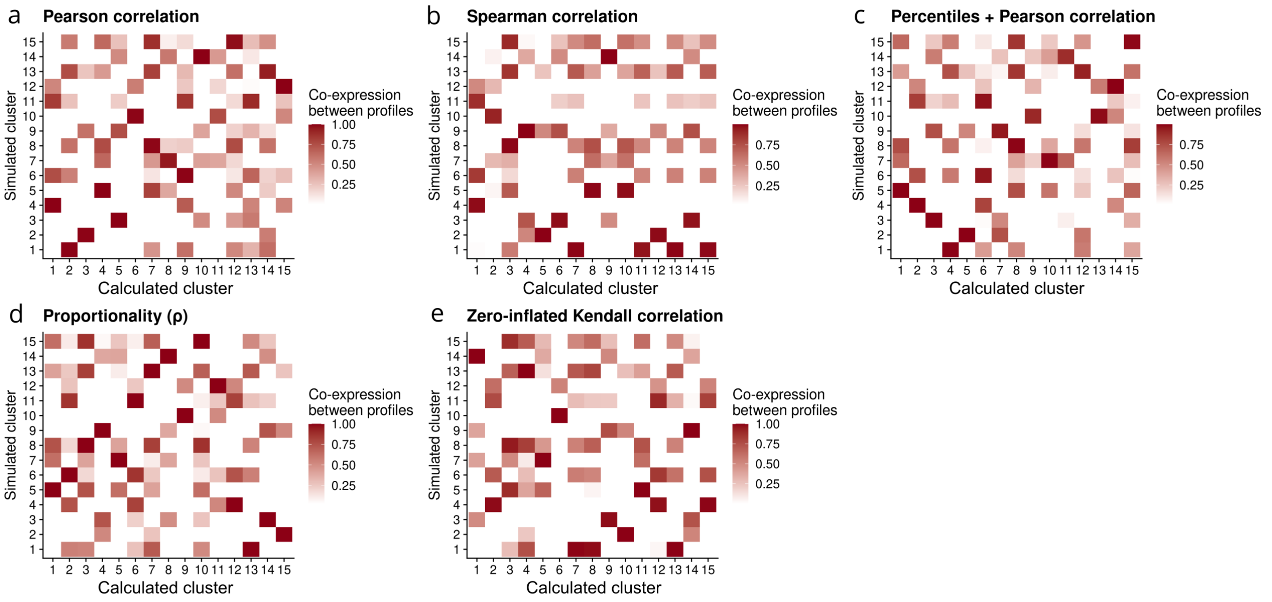

Supplementary Fig. 13 Pearson correlation between mean expression profiles of calculated and simulated clusters. Results shown for the clusters generated using each evaluated metric: **a** Pearson correlation. **b** Spearman correlation. **c** Percentiles + Pearson correlation. **d** Proportionality (ρ). **e** Zero-inflated Kendall correlation. Calculated clusters show no unique match among simulated clusters and were therefore paired with the simulated cluster showing the highest mean expression profile correlation.

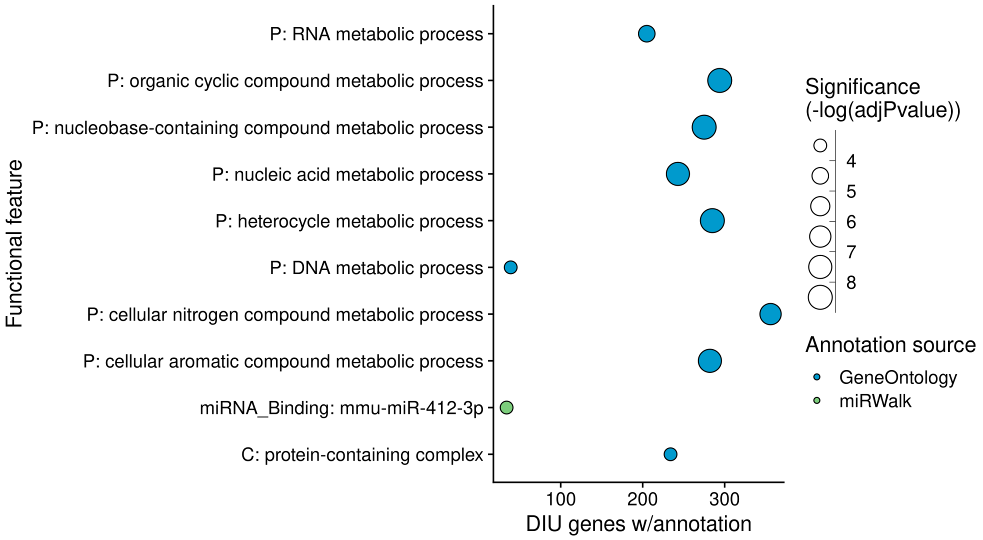

Supplementary Fig. 14 Functional enrichment analysis results for genes with Differential Isoform Usage (DIU) vs genes with Differential Expression (DE) of isoforms. x-axis indicates the total number of test genes (i.e. DIU) including the tested annotation feature. y-axis shows functional features. Dot color represents functional category, dot size represents the significance (-log(adjusted p-value)) obtained in the Fisher's Exact test.

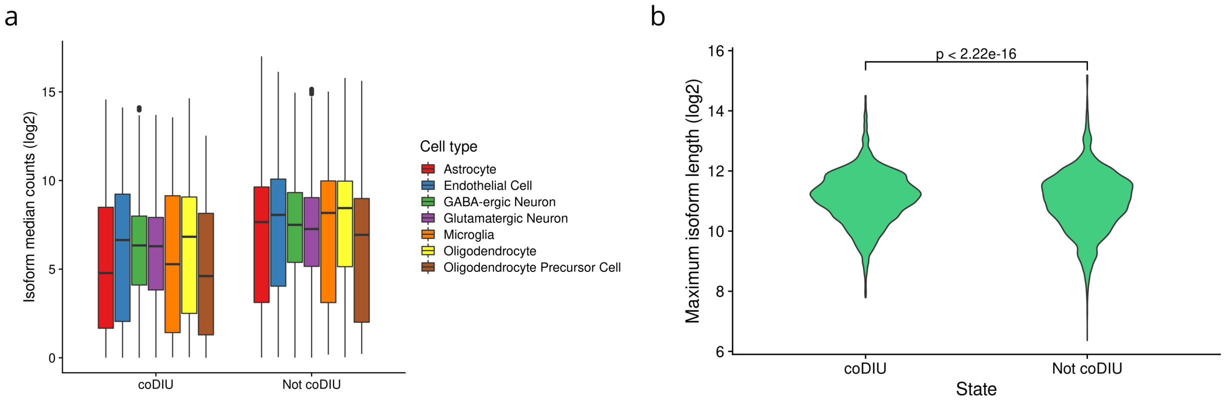

Supplementary Fig. 15 Evaluation of expression and length biases on coDIU genes. a Boxplot of cell type-level median counts (log2) for isoforms belonging to coDIU and not coDIU (i.e. solely DIU) genes. Boxes indicate median (middle line), 25th (Q1) and 75th (Q3) percentiles (box hinges); whiskers represent min = Q1 – 1.5 · Interquartile Range (IQR) and max = Q3 + 1.5 · IQR; dots constitute outliers. **b** Violin plot of isoform length (log2) of longest isoform in coDIU and not coDIU (i.e. isoforms with at least one DE isoform) genes. Significant differences were found between both groups (Wilcoxon's test, two-sided).

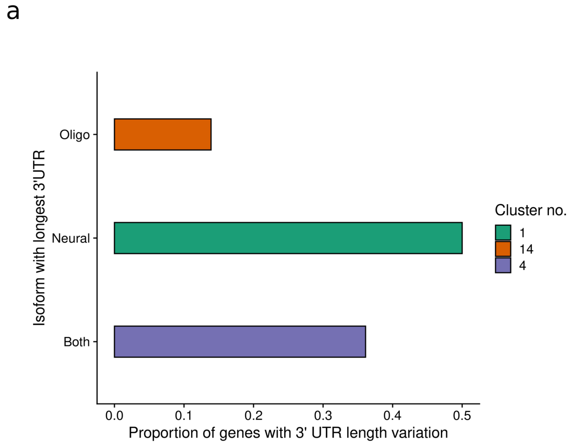

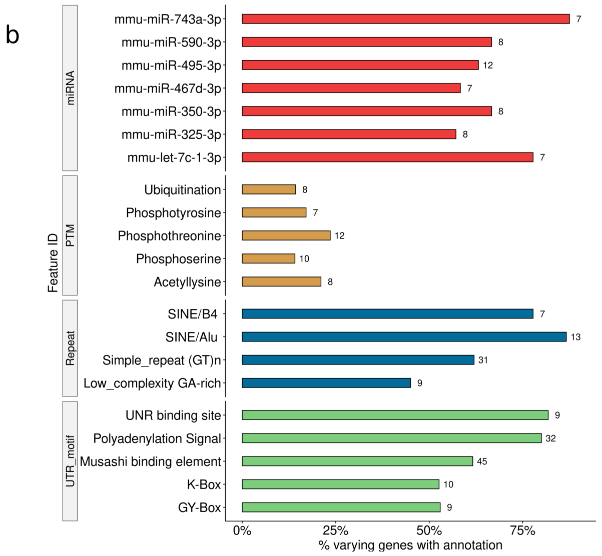

Supplementary Fig. 16 Functional analysis of the 118 coDIU genes detected across neural-oligodendrocyte clusters (no. 1, 4 and 14). a Proportion of coDIU genes with 3'UTR variation across isoforms that have their longest 3'UTR isoform in each of the three analyzed clusters. **b** ID-level FDA results for features in highly-varying functional categories. y-axis shows functional feature categories as boxes and individual features in the axis text. x-axis contains the percentage of genes containing the feature that show functional variation across isoforms. Percentages are shown for the most frequently annotated features in each category. Total coDIU genes with feature indicated by the bar label.

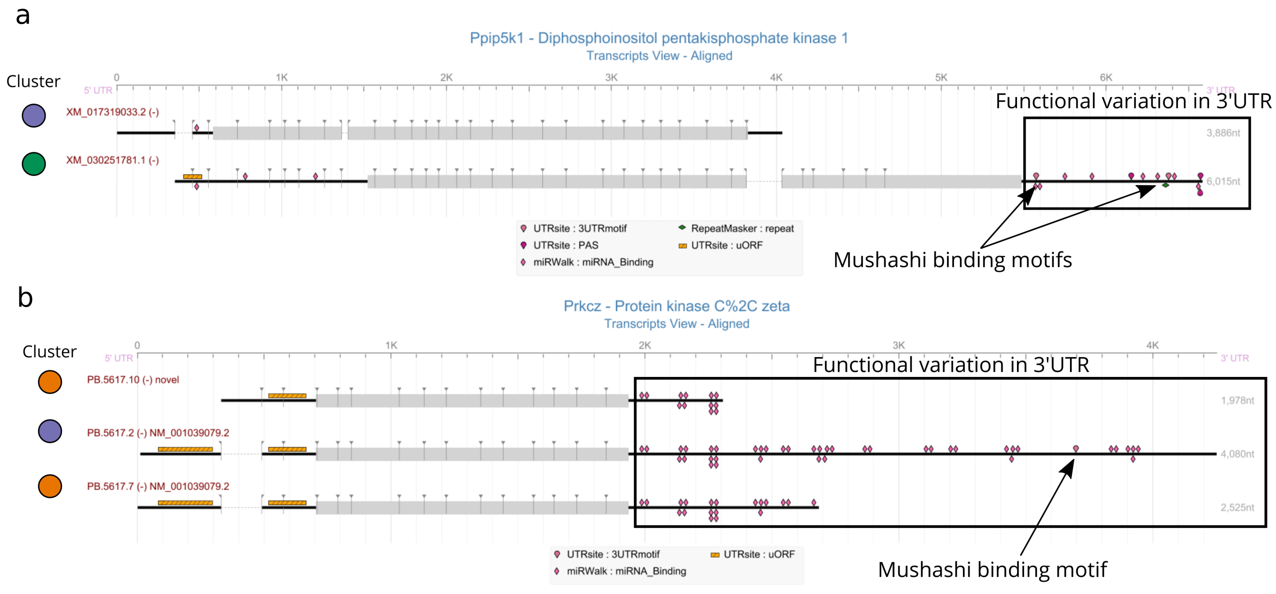

Supplementary Fig. 17 tappAS view of transcript functional annotation for **a** *Ppip5k1* and **b** *Prkcz* isoforms. Cluster assignments for each isoform are indicated by dot color (green: GABA-glutamatergic neuron cluster, orange: oligodendrocyte cluster, purple: expression in both oligodendrocytes and neurons).

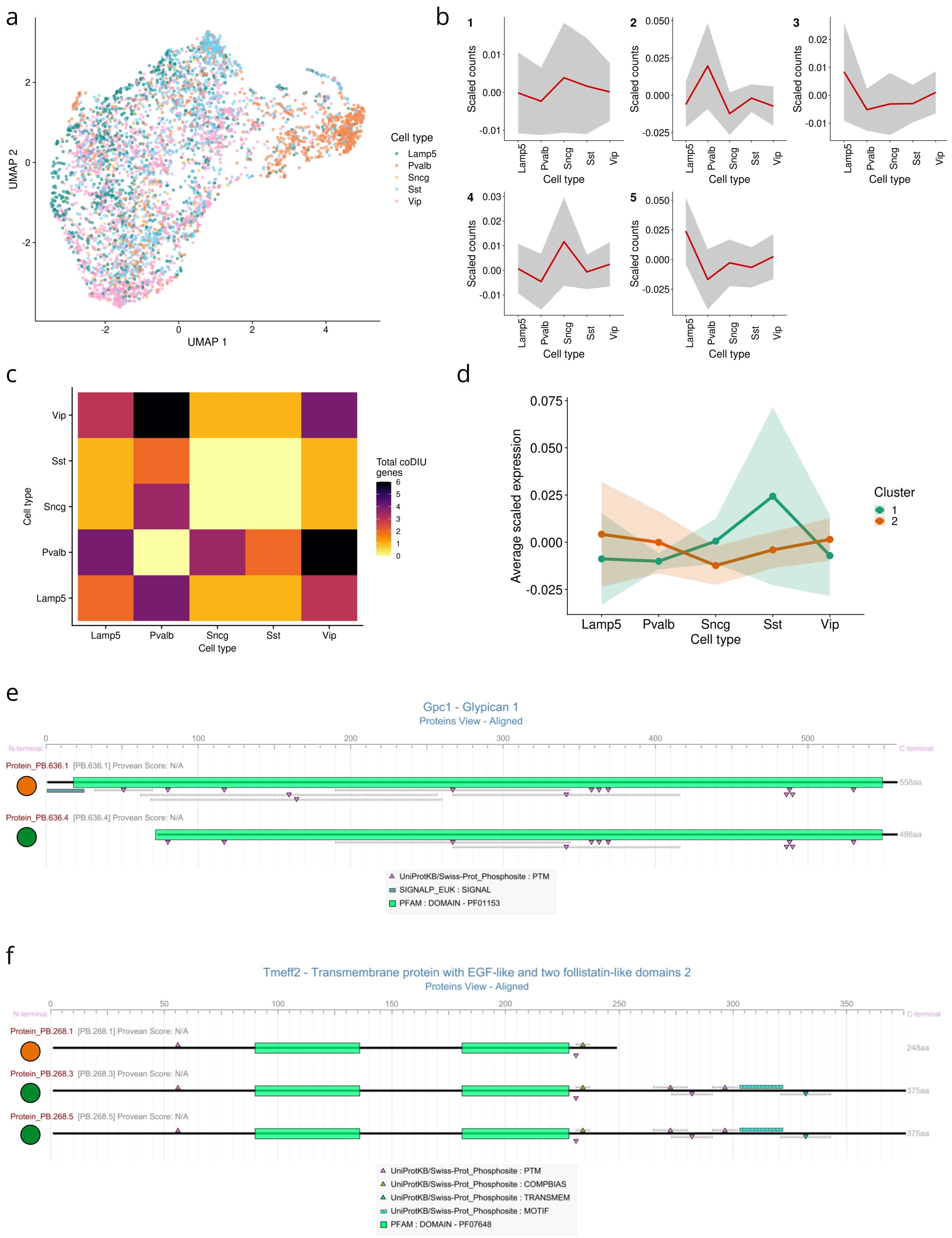

Supplementary Fig. 18 Analysis of coDIU in GABA-ergic neuron subtypes. a UMAP visualization of GABA-ergic neuron subtypes in the Tasic 2018 dataset. **b** Clustering results. For all clustered isoforms, results are averaged at the cell-level and aggregated for each cell type by computing the mean and standard deviations of these cell means. x-axis shows scaled expression (conts), y-axis shows cell subtype labels. **c** Cell type-level coDIU patterns. For each pair of neuron subtypes in the x and y-axis, heatmap color corresponds to the total number of genes found to be co-DIU between them, computed as the sum of coDIU genes detected between all occurrences of clus-

ter pairs showing high expression of isoforms these cell types. Total: 16 coDIU genes. **d** Average expression profile of isoforms from coDIU genes in clusters 1 (7 isoforms) and 2 (13 isoforms). For each cluster, cell-level mean expression (scaled) is computed for all transcripts and cell means are aggregated to obtain a global cell type mean, represented by the lines. Colored areas correspond to cell type mean ± standard deviation.
. tappAS protein view of **e** *Gpc1* and **f** *Tmeff2* genes.

Supplementary Files

Supplementary Data 1: Functional Enrichment results. This file contains a table (TSV format) including the results of intra-category functional enrichment analysis prior to filtering significant GO terms using Revigo semantic similarity. For each functional feature, specified by the *feature_id* column, a one-sided, partially overlapping samples z test was performed to test whether the proportion of coDIU genes annotated with each feature was greater than the proportion of annotated DIU genes. Within each functional feature category (specified by the *source* column), *p*-values were corrected for multiple testing using Benjamini-Hochberg False Discovery Rate (FDR), and results are inclued in the *adjPvalue* column.

Supplementary Note:

Obtaining a high-quality, long read-defined transcriptome

As a part of our study, we have combined bulk PacBio data for neural transcriptome definition and single-cell short-read RNA-Seq data for cell-level isoform quantification. The purpose of this Supplementary Note is to detail the strategy that was used build a high-quality, long read-defined transcriptome using PacBio data, including analytical decisions made during pre-processing, quality control with SQANTI3 and curation using both SQANTI's filtering tools and additional information provided by external supporting data (Figure 1).

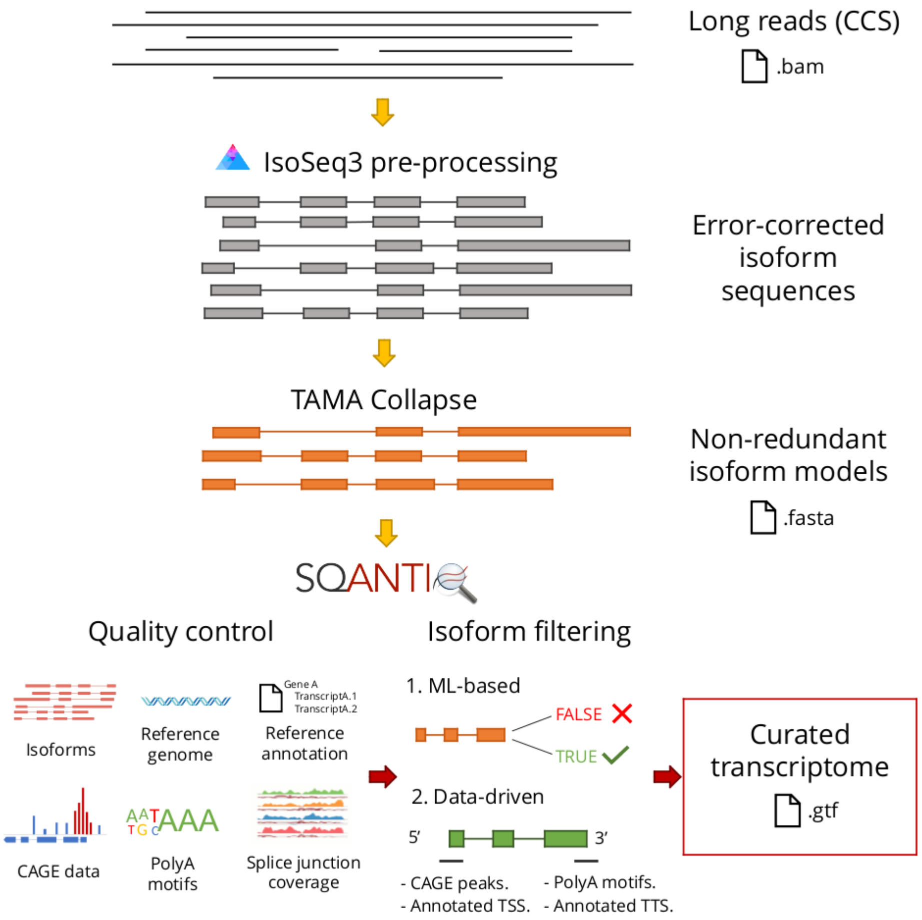

Figure 1: pipeline overview of long read data pre-processing and quality control using SQANTI3.

Long read data and pre-processing

Long-read datasets from mouse hippocampus and cortex[1] were downloaded from ENCODE accessions ENCSR214HSG and ENCSR340GWV, respectively. To obtain a single mouse neural transcriptome, reads from both samples and their replicates (2 replicates/sample) were pooled and pre-processed using the IsoSeq3 pipeline (https://github.com/PacificBiosciences/IsoSeq) (including inter-read correction step, i.e. polishing), with default parameters. After pre-processing, we obtained 178507 isoforms from 20307 genes and characterized the transcriptome using SQANTI3 QC (https://github.com/ConesaLab/SQANTI3) isoform categories (Figure 2A).

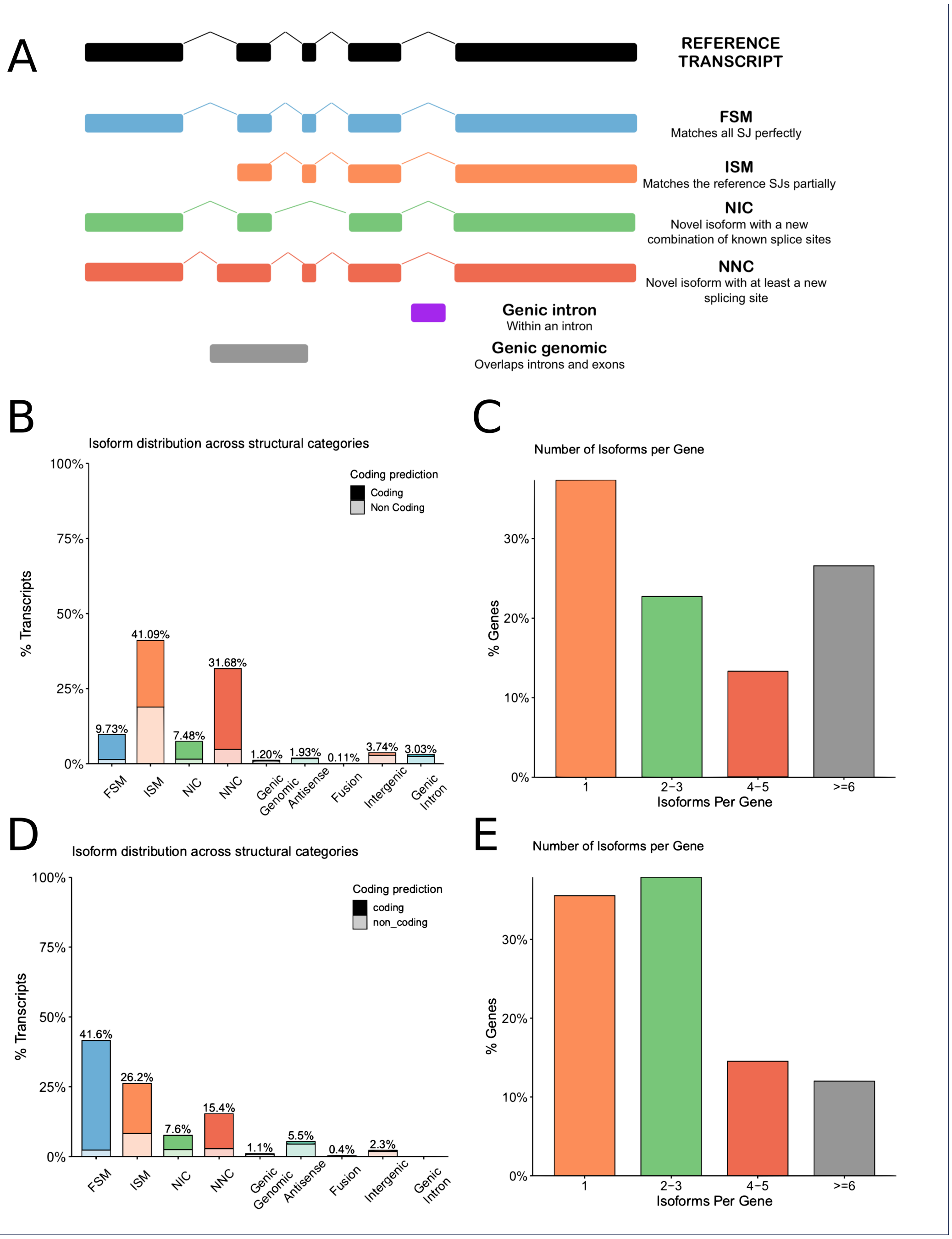

Figure 2: transcriptome overview after pre-processing vs after collapse. A) Description of the main SQANTI3 structural categories (obtained from https://github.com/ConesaLab/SQANTI3). B) Isoform distribution across structural categories for the mouse neural transcriptome after IsoSeq3 pre-processing. C) Number of isoforms per gene after IsoSeq3 pre-processing. C) Isoform distribution across categories after collapse with TAMA. D) Number of isoforms per gene after collapse with TAMA.

Given the dataset's enrichment in Incomplete Splice Matches (ISM, Figure 2B), and to avoid an overestimation of the number of isoforms per gene (Figure 2C), we collapsed redundant isoform models using TAMACollapse[2] (https://github.com/GenomeRIK/tama/wiki/Tama-Collapse). In this manner, we intended to mitigate transcript-level redundancies by merging isoform models that differ due to library preparation and sequencing artifacts, but are very likely to have been generated from the same transcript in the sample. We run TAMACollapse with the following parameters: -c 95 -i 85 -x no_cap -a 10 -m 10 -z 10. This reduced the transcriptome to 93698 isoforms. Collapsing removed a big proportion of ISM isoforms (Figure 2D) and successfully reduced the complexity of our transcriptome (Figure 2E).

Supporting data for long read transcriptome quality control and curation

To perform the quality control of our transcriptome, we run SQANTI3 (https://github.com/ConesaLab/SQANTI3) on the collapsed isoform sequences, using the mouse reference genome (GRCm38.p6) and the RefSeq[3] transcriptome (global release 96, annotation release 108, accessed December 2019). To perform a comprehensive evaluation and subsequent curation of the quality of our isoforms, we also provided supporting data to SQANTI3, namely CAGE peak data, polyA motif sequences and short read splice junction coverage data (Figure 1).

Mouse CAGE peak data were obtained from the FANTOM5 database[4], and used to curate Transcription Start Sites (TSS) for our isoforms. Consistent with previous reports stating that human and mouse polyA motif sequences show high levels of conservation[5,6], we supplied a ranked list of common polyA motif sequences from human (provided with SQANTI3) to curate Transcription Termination Sites (TTS). Finally, to obtain short-read coverage information, and given the lack of matching Illumina data for these long read datasets, we sampled 20 cells

from each of the 7 broad cell types in the Tasic *et al.* dataset[7] and pooled all Illumina reads to generate a pseudo-bulk. We mapped this cell pool to the long read-generated transcriptome (genome version GRCm38.p6) using STAR[8] and the following parameters:

--outFilterType BySJout --outFilterMultimapNmax 20 --alignSJoverhangMin 8 --outFilterMismatchNmax 999 --outFilterMismatchNoverReadLmax 0.04 --alignIntronMin 20 --alignIntronMax 1000.

Transcriptome curation and characterization

In order to curate the long read transcriptome, we defined a strategy for isoform filtering to ensure the quality and realiability of the transcript models (Figure 1). First, we applied SQANTI's random forest classifier[9] (henceforth refered to as ML filter) to perform quality filtering of Novel In Catalog (NIC) and Novel Not in Catalog (NNC) isoforms as well as minority categories such as Antisense, Genic Genomic and Fusion. Next, the above-detailed sources of supplementary data were integrated with information from RefSeq's reference transcriptome (RefSeq release 96, September 2019, genome: GRCm38.p6, annotation: GCF_000001635.26) to remove artifacts belonging to the Full Splice Match (FSM) and Incomplete Splice Match (ISM) categories. To better ilustrate the rational behind this two-step strategy, Figure 3 provides an overview of how each filter modifies transcriptome properties based on SQANTI3 structural categories.

During the first stage of curation, SQANTI's ML filter used 11 isoform-level features, including junction coverage information, to detect false positive isoforms. Of note, this tool was originally designed to detect transcript models that are likely originated by RT-switching, intra-primming and the accumulation of sequencing errors around junctions. Isoforms flagged as artifacts by the classifier were directly removed, reducing our transcriptome from 93698 to 74148 isoforms. In this process, ~40% NIC and ~80% of NNC isoforms were classified as artifacts (Figure 4A), whereas isoforms from other minority categories were subject to equally stringent filtering (Figure 4B). NIC and NNC isoforms labeled as artifacts by SQANTI's ML filter were enriched in low coverage junctions (Figure 4C) and novel splice sites, with the unfiltered transcriptome showing accumulation of non-canonical sites in the NNC category (Figure 4D).

While low support of was especially frequent among novel junctions, isoforms with low-coverage novel splice sites were successfully removed by SQANTI's ML filter (Figure 4E-F).

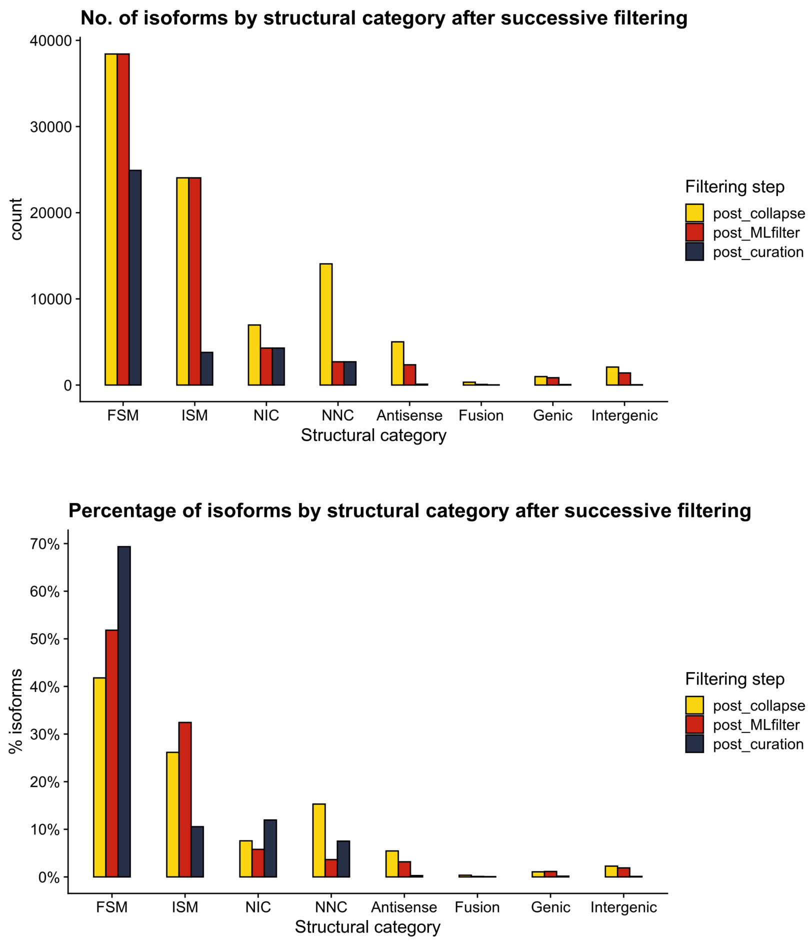

Figure 3: Number and proportion of isoforms within each SQANTI3 structural category in the post-collapse transcriptome and after performing each filtering step.

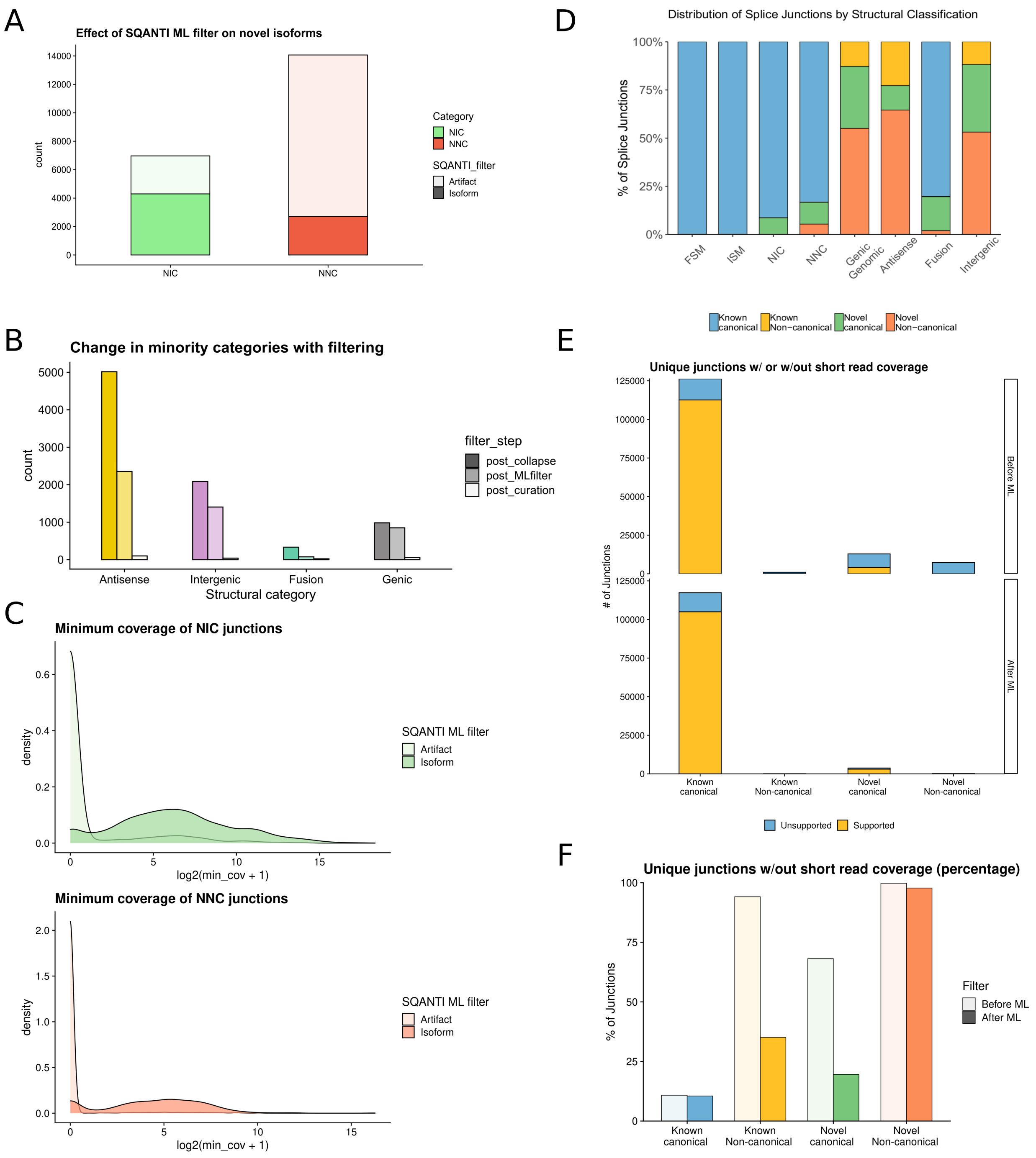

Figure 4: characterization of SQANTI's ML filter. A) Number of NIC and NNC isoformsm flagged as artifacts or isoforms by SQANTI's ML filter. B) Number of isoforms removed after each filtering step in minority structural categories. C) Density distribution of the minimum short-read coverage of junctions, shown for NIC (left) and NNC (rigth) isoforms and stratified by SQANTI's ML filter result. D) Splice junction type (%) present in each SQANTI structural category. E) Number of unique junctions with or without short read coverage for each type of splice junction, before (upper side) and after (lower side) applying SQANTI's ML filter. F) Percentage of unique junctions with or without short read coverage for each type of splice junction, before and after the ML filter.

Next, we established filtering criteria for ISM and FSM isoforms. Transcripts in these two categories are used as true positives in SQANTI's ML filter, and therefore will be preserved without evaluating their reliability. However, while these categories solely present know (and therefore validated) junctions, they often incorporate 3' and 5' end variability, which can lead to the detection of multiple FSM and/or ISM isoforms per reference transcript, i.e. isoforms for whom all junctions have been previously reported by the reference annotation, but differing in their start and/or end positions. We named this phenomenon "redundancy". Importantly, redundancy can be the result of true TSS and TTS diversity or stem from library preparation artifacts such as RNA degradation or intra-priming, resulting in false positive novel TSS and TTS. In our transcriptome, high levels of redundancy were observed for both FSM (Figure 5A) and ISM (Figure 5B). To mitigate this, the amount of redundancy that can be confidently preserved was evaluated using CAGE peak data (5' end) and polyA motif information (3' end).

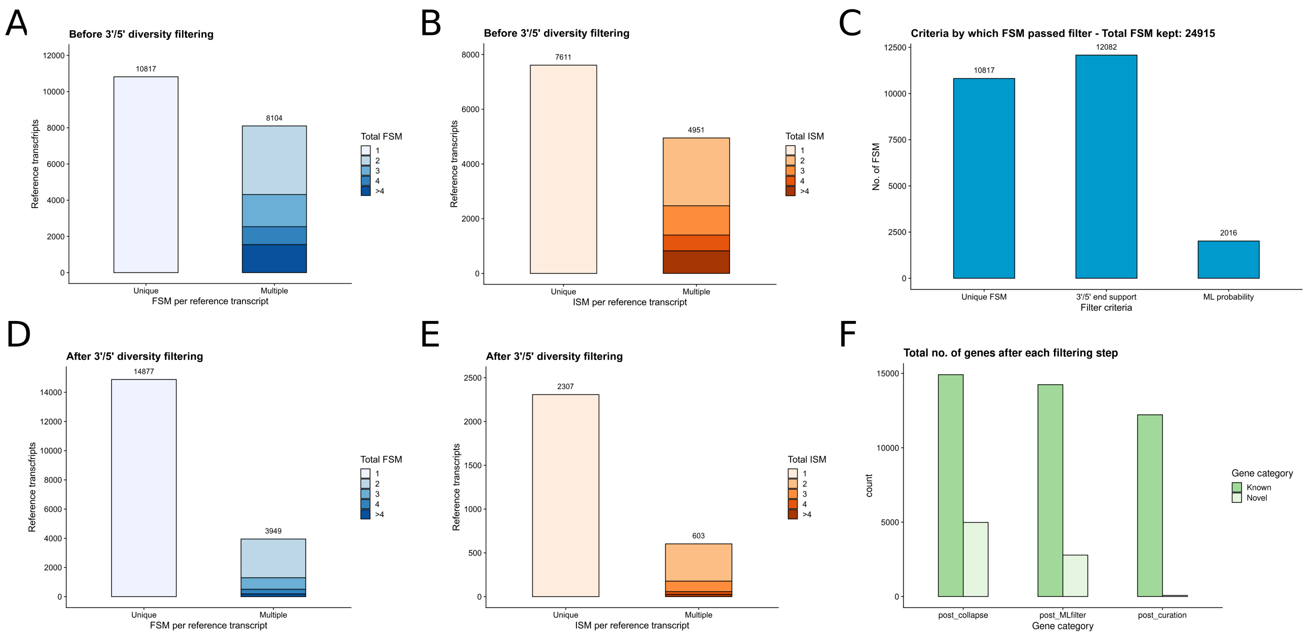

Figure 5: effect of multiple-criteria filtering on the long read transcriptome. A) and B) Redundancy levels for FSM (A) and ISM (B) before filtering based on CAGE and polyA data. C) FSM count stratified by the criteria used to included the isoform in the transcriptome. D) and E) Redundancy levels for FSM (D) and ISM (E) after filtering. F) Number of known and novel genes after collapse, and after each filtering step.

For FSM isoforms, we evaluated the suitability of preserving an isoform in the transcriptome according to three criteria, in the following order of priority: a) detection of a reference-compatible FSM transcript, in cases where there is no redundancy and even if no support is found; b) transcripts with support for TSS and TTS to preserve only reliable FSM, if there is redundancy; c) selection of the FSM that best matches the reference transcript, when there was redundancy but no 3'/5' end support for any of the FSM found. For ISM, given that missing junctions from matching reference transcripts increases the probability of false epositive TSS/TTS, we only applied criteria (b). These rules and their effect on the transcriptome will be described in more detail below.

First, transcripts that constituted the only matching FSM of a given reference transcript, i.e. unique-FSM cases, were automatically preserved, independently of CAGE/polyA support of their TSS and TTS. In this manner, we prioritize evidence of detection of junction combinations present in reference transcripts over TSS/TTS definition accuracy. Next, we evaluated TSS and TTS end positions to discriminate false positive isoforms in multiple-FSM cases, i.e. those where multiple FSM per reference transcript were detected. Regarding the 5' end, we kept isoforms whose TSS was included within or situated up to 50bp downstream of a CAGE peak, to account for degradation, and whose 3' end region included a polyA motif no further than 50bp upstream of the TTS. Alternatively, isoforms lacking CAGE or polyA support were included in the transcriptome if their TSS matched or was situated up to 50bp downstream of an annotated TSS, and their TTS also matched or was within ±50bp of an annotated TTS. In this manner, we consider both CAGE/polyA support and the reference transcriptome for curation, and include all possible combinations of these two sources in our transcriptome. For instance, an isoform will be included if it contains a novel TSS supported by CAGE, and its TTS is close enough to that of the reference transcript. ISM isoforms were similarly filtered according to polyA/CAGE and annotated TSS or TTS evidence. Finally, for multiple-FSM cases where no FSM met the TSS/TTS requirements, we devised a strategy to select the best match among detected isoforms. In this manner, and similarly to the unique FSM scenario, we prioritize evidence of detection of a reference-annotated combination of junctions. We preserved the FSM transcript with the highest random forest probability score, as output by the ML filter in SQANTI.

As a result of curation, 35700 isoforms from 12183 genes were preserved. All in all, this filtering strategy not only managed to enrich the transcriptome in FSM (24915 total FSM, ~67%, Figure 3), but also pointed out the importance of combining multiple criteria to prevent losing relevant FSM transcripts that would have been lost based solely on 3'/5' end support, with ~50% of FSM in our transcriptome being preserved based on unique FSM or best match criteria (Figure 5C). Furthermore, filtering effectively reduced redundancy in both FSM (Figure 5D) and ISM (Figure 5E), simultaneously retaining some multiple matches for both categories. Part of the 3'/5' diversity in the long read-defined transcriptome could therefore be attributed to true TSS and TTS, however, no isoforms from novel genes were preserved, given their lack of 3'/5' end support (Figure 5F) and thus our inability to tell true isoforms apart from artifacts.

Since we combined different data sources for filtering, we decided to inspect whether supporting data or agreement with the reference annotation (or both) had been the reasons for inclusion in the transcriptome. In doing so, we found only moderate levels of agreement between CAGE and polyA data and the reference annotation, highlighting the importance of supporting data for transcriptome curation. In the case of FSM, more than 50% of validated TSS and TTS could only be confirmed using CAGE and polyA motif data (Figure 6A), and this percentage was even higher for some ISM sub-categories (Figure 6B). For instance, internal fragments (missing junctions at both the 3' and 5' ends in comparison to the matching reference transcript) could only be validated using CAGE or polyA information. All in all, these analyses suggest that reference transcripts may contain multiple, unannotated TSS and TTS that are can only be observed using long reads.

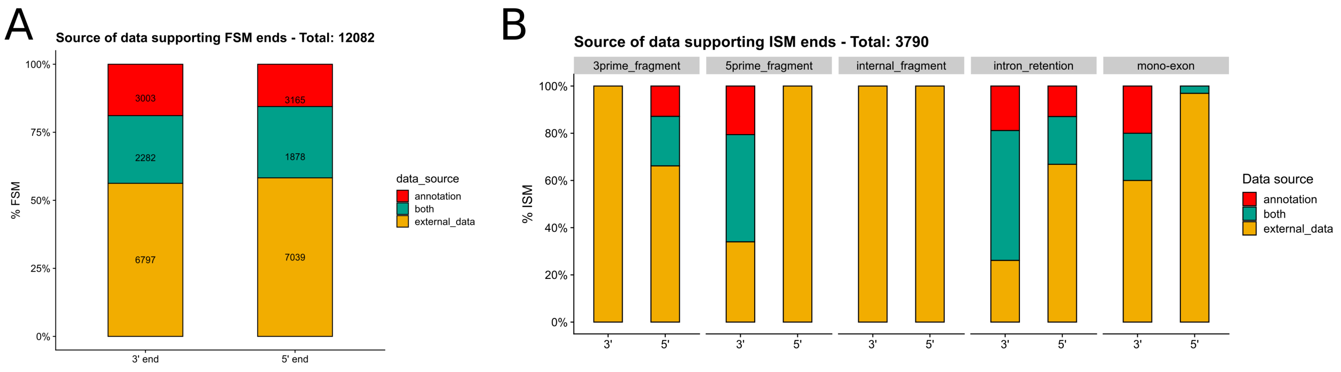

Figure 6: source of data guiding isoform inclusion during end-support filtering. A) Included FSM % supported by external (i.e. supporting) data, the reference annotation or both, for 3' and 5' end support. B) Included FSM % supported by each supporting data type, stratified by ISM sub-category.

In addition to the former, we performed a recovery process for genes for which multiple isoforms had been detected using long reads, but for which non of these long read-defined isoforms passed the TSS/TTS filtering. In these cases, reads compatible with multiple reference transcripts may have been found, even if long read data was not reliable enough for isoform model reconstruction. We focused on removed genes that had multiple FSM isoforms associated to two or more reference transcripts, and incorporated their associated reference transcripts from RefSeq to the transcriptome, while discarding the unsupported long read-defined isoforms. Consequently, we added 1286 missing isoforms and 509 genes for which there was evidence of alternative isoform expression, even if their isoforms could not be confidently defined using long reads. All in all, 36986 isoforms from 12692 genes were included in our final, curated transcriptome.

It should be noted that, for minority categories, checking for CAGE/polyA and annotation support resulted in the removal of practically all isoforms (Figure 4B). Given the difficulty in establishing the reliability of the remaining isoforms, or the relevance of CAGE/polyA data for this task, the few transcripts from categories that remained were excluded from the transcriptome.

Functional annotation of long read-defined isoforms

Recent studies by several research groups, including our own, have made a strong case for the advantages of coupling expression-level with functional analysis when it comes to fully understanding alternative splicing regulation[10,11]. To this end, we decided to complete our long read-defined transcript models with isoform-level functional information using IsoAnnotLite (https://isoannot.tappas.org/isoannot-lite/). IsoAnnotLite can incorporate functional information from sequence-based predictors and biological databases into any long read-defined transcriptome, as long as an already-available, compatible functional annotation -i.e. the ones available within the tappAS framework[10]- is provided. The set of compatible functional features (such as domains, motifs or binding regions for other molecular elements) is diverse, and encompasses both the transcript and protein isoform levels. All in all, we aimed to use the co-expression analysis pipeline in the *acorde* R package (https://github.com/ConesaLab/acorde) to predict the changes in inclusion of functional

features encoded by isoform-level co-expression across cell types, and therefore the potential of co-selected alternative isoforms to exert different biological functions.

Briefly, IsoAnnotLite requires four inputs for functional annotation: SQANTI3 outputs **classification.txt*, **junctions.txt* and **corrected.gtf* files, each of them containing information regarding long read-defined transcripts, and a tappAS-compatible GFF3 file containing functional annotations. Functional features are then transferred from the GFF3 source annotation file to the GTF target annotation file. We run IsoAnnotLite v2.6 using SQANTI3 output files for our transcriptome, and a functional annotation GFF3 file generated after combining the mouse RefSeq functional annotation available in tappAS (RefSeq78), and a comprehensively-annotated mouse neural transcriptome belonging to neural progenitors[10]. We selected several functional categories from each of them, as detailed in Table 1. Once the input files are provided, IsoAnnotLite performs what we refer to as positional transfer of features. First, functional information that is positionally defined within isoforms in the GFF3 source file is translated to its corresponding genomic position. Then, functional feature information is mapped to the unannotated isoforms in the target GTF if the feature's genomic positions are included within any of the transcript's exons. The process is performed for both transcript and protein-level annotations, as well as for genomic functional information such as Gene Ontology (GO) terms, KEGG pathway information, if present. We run IsoAnnotLite (v2.6) using the following non-default parameters: *-gff3* (provides the combined GFF3 annotation file) and *-novel.*

As a result, 35028 isoforms in the GTF target annotation file (94.71% of 36986 isoforms in our transcriptome) were annotated, meaning that at least one functional feature could be successfully transferred from the source annotation file. The remaining 696 isoforms received no functional information due to the fact that the corresponding gene was not found in the GFF3 file, however, these represent but 1.88% of the long read-defined isoforms, meaning that feature transference was effective even though the reference GFF3 annotation files used did not exactly correspond to the same mouse samples, or even the same reference transcriptome version, namely RefSeq96 was used as the reference annotation for transcriptome construction and quality control using PacBio ENCODE data, while tappAS' mouse GFF3 functional annotation belonged to RefSeq version 78.

Regarding the comprehensiveness of the annotation process, IsoAnnotLite reported that ~47% of transcripts in the target GTF had the same associated reference transcript as one of the isoforms in the GFF3 reference annotation, which meant total agreement between their splice junctions and therefore ensured perfect possitional transference of features in the case of these isoforms. Even more reassuringly, feature transference in the 3’ and 5’ UTRs (regions that may differ even when the reference associated transcript is the same) is completed by IsoAnnotLite’s algorithm if the alternative UTR regions are included in other reference GFF3 transcripts, and contain functional features. For the remaining long read-defined transcripts, transference depended on the differences among alternative isoforms from the same genes present in the target and reference functional annotations, and was therefore expected to vary across genes. Consequently, the total number of functional features recovered was diverse depending on the functional category under evaluation (Table 1). It should be noted, however, that these numbers additionally rely on the extent to which these categories were originally present in the reference functional annotation files used, with less frequently annotated categories being often rare in their source GFF3 file (for instance, intramembrane protein regions). A complementary explanation has to do with the fact that the mouse NSC time series transcriptome was considerably more condition-specific than RefSeq, decreasing the possilibty to match the genes and isoforms included in the target GTF, and making transference challenging for categories extracted from it, such as RBPs and NLS.

Source functional database	Feature category	No. of annotated functional features	Source GFF3 annotation file
COILS	COILED	1184	Mouse NSC time series
CORUM	Complex	197	Mouse NSC time series
GeneOntology	C	32163	Mouse RefSeq78
GeneOntology	F	30144	Mouse RefSeq78
GeneOntology	P	30245	Mouse RefSeq78
KEGG	Pathway	2652	Mouse RefSeq78
MetaCyc	Pathway	1823	Mouse RefSeq78
miRWalk	miRNA binding	186755	Mouse RefSeq78
MOBIDB LITE	DISORDER	2732	Mouse NSC time series
NLS mapper	MOTIF	1020	Mouse NSC time series
NMD prediction	NMD	268	Mouse RefSeq78
PAR-clip data	RBP binding	3000	Mouse NSC time series
PFAM	CLAN	23526	Mouse RefSeq78
PFAM	DOMAIN	50736	Mouse RefSeq78
Reactome	Pathway	11988	Mouse RefSeq78
RepeatMasker	Repeat	35075	Mouse RefSeq78
scanForMotifs	3UTRmotif	4078	Mouse NSC time series
SIGNALP EUK	SIGNAL PEPTIDE	2641	Mouse NSC time series
TMHMM	TRANSMEMBRANE	1379	Mouse NSC time series
UniProtKB/Swiss-Prot	ACTIVE SITE	4790	Mouse NSC time series
UniProtKB/Swiss-Prot	BINDING	32126	Mouse NSC time series
UniProtKB/Swiss-Prot	COILED	3201	Mouse NSC time series
UniProtKB/Swiss-Prot	COMPBIAS	6392	Mouse NSC time series
UniProtKB/Swiss-Prot	INTRAMEMBRANE	385	Mouse NSC time series
UniProtKB/Swiss-Prot	MOTIF	15126	Mouse NSC time series
UniProtKB/Swiss-Prot	PTM	237128	Mouse NSC time series
UniProtKB/Swiss-Prot	TRANSMEMBRANE	19293	Mouse NSC time series
UTRsite	3UTRmotif	44013	Mouse RefSeq78
UTRsite	5UTRmotif	623	Mouse RefSeq78
UTRsite	PAS	8787	Mouse RefSeq78
UTRsite	uORF	13370	Mouse RefSeq78

Table 1: Summary of IsoAnnotLite functional feature transference. *Source functional database* refers to the original source of the functional information, which can be either a biological database or a sequence-based prediction tool. *Feature categories* are broad terms under which multiple entries of functional features encoding similar functions are classified. *Functional features* are the specific motifs, domains, regions, etc. annotated within the transcriptome's isoforms.

Source GFF3 annotation file refers to the functionally annotated transcriptome from which the feature information for each of the functional categories was obtained, and then passed on to IsoAnnotLite for feature transference. *NLS: nuclear localization signal; NMD: nonsense-mediated decay; NSC: Neural Stem Cells; PTM: post-translational modification; PAS: polyadenylation signal; RBP: RNA binding protein; uORF: upstream open reading frame.*

References

1. Wyman, D. *et al.* A technology-agnostic long-read analysis pipeline for transcriptome discovery and quantification. *bioRxiv* (2019) doi:10.1101/672931.

2. Kuo, R. I. *et al.* Illuminating the dark side of the human transcriptome with long read transcript sequencing. *BMC Genomics* **21**, 1–22 (2020).

3. O'Leary, N. A. *et al.* Reference sequence (RefSeq) database at NCBI: Current status, taxonomic expansion, and functional annotation. *Nucleic Acids Res.* **44**, D733–D745 (2016).

4. Forrest, A. R. R. *et al.* A promoter-level mammalian expression atlas. *Nature* **507**, 462–470 (2014).

5. Tian, B., Hu, J., Zhang, H. & Lutz, C. S. A large-scale analysis of mRNA polyadenylation of human and mouse genes. *Nucleic Acids Res.* **33**, 201–212 (2005).

6. Wang, R., Zheng, D., Yehia, G. & Tian, B. A compendium of conserved cleavage and polyadenylation events in mammalian genes. *Genome Res.* **28**, 1427–1441 (2018).

7. Tasic, B. *et al.* Adult mouse cortical cell taxonomy revealed by single cell transcriptomics. *Nat. Neurosci.* **19**, 335–346 (2016).

8. Dobin, A. *et al.* STAR: Ultrafast universal RNA-seq aligner. *Bioinformatics* **29**, 15–21 (2013).

9. Tardaguila, M. *et al.* SQANTI: extensive characterization of long-read transcript sequences for quality control in full-length transcriptome identification and quantification. *Genome Res.* **28**, 396–411 (2018).

10. de la Fuente, L. *et al.* tappAS: a comprehensive computational framework for the analysis of the functional impact of differential splicing. *Genome Biol.* **21**, 119 (2020).

11. Vitting-Seerup, K. & Sandelin, A. IsoformSwitchAnalyzeR: analysis of changes in genome-wide patterns of alternative splicing and its functional consequences. *Bioinformatics* **35**, 4469–4471 (2019).
