# Supplementary material for: acorde unravels functionally interpretable networks of isoform co-usage from single cell data
Source: Nat Commun. 2022 Apr 5;13:1828. doi: 10.1038/s41467-022-29497-w (PMC8983708; doi:10.1038/s41467-022-29497-w)
Supplement: Supplementary file 2 — Reporting Summary [file 41467_2022_29497_MOESM2_ESM.pdf]

nature research

Corresponding author(s): Ana Conesa

Last updated by author(s): 28-05-2021

Reporting Summary

Nature Research wishes to improve the reproducibility of the work that we publish. This form provides structure for consistency and transparency in reporting. For further information on Nature Research policies, see our Editorial Policies and the Editorial Policy Checklist.

Statistics

For all statistical analyses, confirm that the following items are present in the figure legend, table legend, main text, or Methods section.

n/a	Confirmed	
☐	☒	The exact sample size (n) for each experimental group/condition, given as a discrete number and unit of measurement
☒	☐	A statement on whether measurements were taken from distinct samples or whether the same sample was measured repeatedly
☐	☒	The statistical test(s) used AND whether they are one- or two-sided<br>*Only common tests should be described solely by name; describe more complex techniques in the Methods section.*
☐	☒	A description of all covariates tested
☐	☒	A description of any assumptions or corrections, such as tests of normality and adjustment for multiple comparisons
☐	☒	A full description of the statistical parameters including central tendency (e.g. means) or other basic estimates (e.g. regression coefficient) AND variation (e.g. standard deviation) or associated estimates of uncertainty (e.g. confidence intervals)
☐	☒	For null hypothesis testing, the test statistic (e.g. F, t, r) with confidence intervals, effect sizes, degrees of freedom and P value noted<br>*Give P values as exact values whenever suitable.*
☒	☐	For Bayesian analysis, information on the choice of priors and Markov chain Monte Carlo settings
☐	☒	For hierarchical and complex designs, identification of the appropriate level for tests and full reporting of outcomes
☐	☒	Estimates of effect sizes (e.g. Cohen's d, Pearson's r), indicating how they were calculated

Our web collection on statistics for biologists contains articles on many of the points above.

Software and code

Policy information about availability of computer code

Data collection	No software was used for data collection.
Data analysis	PacBio data processing: IsoSeq3 (v3.2.2, https://github.com/PacificBiosciences/IsoSeq), TAMACollapse (v1.0, https://github.com/GenomeRIK/tama), SQANTI3 QC (v1.0, https://github.com/ConesaLab/SQANTI3), SQANTI ML filter (v1.0, https://github.com/ConesaLab/SQANTI3)<br>Functional annotation: IsoAnnotLite (v2.6, https://isoannot.tappas.org/isoannot-lite/)<br>Isoform quantification: STAR (v2.7.3a, https://github.com/alexdobin/STAR), RSEM (v1.3.0, https://github.com/deweylab/RSEM).<br>Quality control: NOISeq (2.36, https://www.bioconductor.org/packages/release/bioc/html/NOISeq.html).<br>Isoform expression analysis: acorde R package (v1.0). https://github.com/ConesaLab/acorde. Functions from other R packages employed during acorde development are described in Methods and in the package's documentation.<br>Functional analysis: tappAS (v1.0.7, https://github.com/ConesaLab/tappAS).

For manuscripts utilizing custom algorithms or software that are central to the research but not yet described in published literature, software must be made available to editors and reviewers. We strongly encourage code deposition in a community repository (e.g. GitHub). See the Nature Research guidelines for submitting code & software for further information.

Data

Policy information about availability of data

All manuscripts must include a data availability statement. This statement should provide the following information, where applicable:
- Accession codes, unique identifiers, or web links for publicly available datasets
- A list of figures that have associated raw data
- A description of any restrictions on data availability

Reference genome and transcriptomes for mouse assembly were downloaded from the RefSeq database, global release 96, annotation release 108 (september

2019). Genome accesion: GRCm38.p6. Transcriptome accession: GCF_000001635.26.
Single-cell, short-read Illumina data by Tasic et al. (2016) was downloaded from Sequence Read Archive accession SRP061902.
Single-cell, short-read Illumina data by Tasic et al. (2018) was downloaded from Sequence Read Archive accession SRP150473 .
Long-read datasets from mouse hippocampus and cortex from Wyman et al. (bioRxiv, 2019) were downloaded from ENCODE accessions ENCSR214HSG and ENCSR340GWV, respectively.

Field-specific reporting

Please select the one below that is the best fit for your research. If you are not sure, read the appropriate sections before making your selection.

- [x] Life sciences
- [ ] Behavioural & social sciences
- [ ] Ecological, evolutionary & environmental sciences

For a reference copy of the document with all sections, see nature.com/documents/nr-reporting-summary-flat.pdf

Life sciences study design

All studies must disclose on these points even when the disclosure is negative.

Sample size

No sample size calculations performed because public data was employed in the study. Regarding the number of datasets, we decided to use a first dataset (Tasic et al. 2016) to develop and illustrate our method, and included a second dataset (Tasic et al. 2018) to demonstrate that the method is generalizable to any dataset.

Data exclusions

No data were excluded from the analysis.

Replication

Our algorithm considers cell-level measurements to be technical replicates of the same cell state, ensuring the robustness of our findings.
Since the study does not include experimental results, replication does not apply in this case.
For DE analysis, we successfully performed 50 replications of random cell selection + differential expression in order to ensure a robust downstream analysis.

Randomization

Randomization is not relevant to the study, given that sample (i.e. cell) grouping into cell types cannot be randomized.

Blinding

Blinding is not relevant to the study, given that sample ID, i.e. cell - cell type correspondence, is essential to the data analysis process in the present study. In addition, cell type-specific information is used to make informed decisions during the analysis, e.g. downsampling + DE in the case of very abundant cell types (which was performed x50 to ensure robustness). Finally, our computational method is designed to be agnostic to sample IDs (i.e. cell and cell type labels), removing the necessity for blinding.

Reporting for specific materials, systems and methods

We require information from authors about some types of materials, experimental systems and methods used in many studies. Here, indicate whether each material, system or method listed is relevant to your study. If you are not sure if a list item applies to your research, read the appropriate section before selecting a response.

Materials & experimental systems

n/a	Involved in the study
x	Antibodies
x	Eukaryotic cell lines
x	Palaeontology and archaeology
x	Animals and other organisms
x	Human research participants
x	Clinical data
x	Dual use research of concern

Methods

n/a	Involved in the study
x	ChIP-seq
x	Flow cytometry
x	MRI-based neuroimaging
