# Supplementary material for: acorde unravels functionally interpretable networks of isoform co-usage from single cell data
Source: Nat Commun. 2022 Apr 5;13:1828. doi: 10.1038/s41467-022-29497-w (PMC8983708; doi:10.1038/s41467-022-29497-w)
Supplement: Supplementary file 4 — Description of Additional Supplementary Files [file 41467_2022_29497_MOESM4_ESM.pdf]

**Title: Supplementary Data 1:**

**Description: Functional Enrichment results.** This file contains a table (TSV format) including the results of intra-category functional enrichment analysis prior to filtering significant GO terms using Revigo semantic similarity. For each functional feature, specified by the *feature_id* column, a one-sided, partially overlapping samples z test was performed to test whether the proportion of co-DIU genes annotated with each feature was greater than the proportion of annotated DIU genes. Within each functional feature category (specified by the *source* column), *p*-values were corrected for multiple testing using Benjamini-Hochberg False Discovery Rate (FDR), and results are inclued in the *adjPvalue* column.
